# Isolation, Characterization and Anticancer Potential of Cytotoxic Triterpenes from *Betula utilis* Bark

**DOI:** 10.1371/journal.pone.0159430

**Published:** 2016-07-25

**Authors:** Tripti Mishra, Rakesh Kumar Arya, Sanjeev Meena, Pushpa Joshi, Mahesh Pal, Baleshwar Meena, D. K. Upreti, T. S. Rana, Dipak Datta

**Affiliations:** 1 Phytochemistry Division, CSIR-National Botanical Research Institute, Lucknow, 226 001, India; 2 Biochemistry Division, CSIR-Central Drug Research Institute (CDRI), Lucknow, 226031, India; 3 Department of chemistry, D.S.B. Campus Kumaun University, Nainital, 263002, India; 4 Plant Diversity, Systematics and Herbarium Division, CSIR-National Botanical Research Institute, Lucknow, 226001, India; University of Nebraska Medical Center, UNITED STATES

## Abstract

*Betula utilis*, also known as Himalayan silver birch has been used as a traditional medicine for many health ailments like inflammatation, HIV, renal and bladder disorders as well as many cancers from ages. Here, we performed bio-guided fractionation of *Betula utilis* Bark (BUB), in which it was extracted in methanol and fractionated with hexane, ethyl acetate, chloroform, n-butanol and water. All six fractions were evaluated for their *in-vitro* anticancer activity in nine different cancer cell lines and ethyl acetate fraction was found to be one of the most potent fractions in terms of inducing cytotoxic activity against various cancer cell lines. By utilizing column chromatography, six triterpenes namely betulin, betulinic acid, lupeol, ursolic acid (UA), oleanolic acid and β-amyrin have been isolated from the ethyl acetate extract of BUB and structures of these compounds were unraveled by spectroscopic methods. β-amyrin and UA were isolated for the first time from *Betula utilis*. Isolated triterpenes were tested for *in-vitro* cytotoxic activity against six different cancer cell lines where UA was found to be selective for breast cancer cells over non-tumorigenic breast epithelial cells (MCF 10A). Tumor cell selective apoptotic action of UA was mainly attributed due to the activation of extrinsic apoptosis pathway via up regulation of DR4, DR5 and PARP cleavage in MCF-7 cells over non-tumorigenic MCF-10A cells. Moreover, UA mediated intracellular ROS generation and mitochondrial membrane potential disruption also play a key role for its anti cancer effect. UA also inhibits breast cancer migration. Altogether, we discovered novel source of UA having potent tumor cell specific cytotoxic property, indicating its therapeutic potential against breast cancer.

## Introduction

*Betula utilis* (Himalayan silver birch, Betulaceae), is a moderate-sized tree, which attains a height up to 20m. It is commonly known as *Bhojpatra* and found in the high altitudes of Himalayas. The bark is smooth shining, reddish white or white, with white horizontal lenticels. The outer bark consists of numerous thin papery layers, exfoliating in broad horizontal rolls. The inner cortex is red and moist. The stem bark is used in the ayurvredic system of medicine for treatment of various diseases as wound healing, convulsions, leprosy [1–3], it also has antiseptic, carminative and contraceptive properties [4]. The bark contains several chemical compounds like betulin, lupeol, oleanolic acid, acetyloleanolic acid, betulinic acid, lupenone, sitosterol, methyle betulonate, methyl betulate and karachic acid. [4–6] Besides, Himalayan silver birch has potential applications in the skin and cosmetic industries [7–9]. *Betula utilis* bark was found effective against human pathogenic bacteria [10]. Fatty acid constituents present in the *Betula utilis* bark are linoleic (17.66%), myristic (15.9%), palmitic (9.09%), Oleic (11.30%)[11]. Essential oil of *Betula utilis* bark shows presence of geranic acid, seleneol, Linalool, Sesquiphellendrene, Champacol, 1,8-cineol. Essential oil of Betula utilis bark has a strong antimicrobial activity against the fungus *Candida albicans* and Gram (+) and Gram (-) human pathogenic bacteria [12].

Anticancer potential of an extract or molecule largely depends on the generation of reactive oxygen species (ROS), which subsequently leads to the disruption of mitochondrial membrane potential resulting apoptosis of tumor cells [13]. The present work deals with the primary screening of cytotoxic activity of extracts, isolation of bioactive molecules from fraction having significant cytotoxic activity, structure elucidation of all isolated compounds and identification of compound and its mechanism of action responsible for cytotoxic activity of *Betula utilis* bark of western Himalaya.

## Results and Discussion

### Anticancer screening of *Betula utilis* bark extracts for cytotoxic activity

**Fig 1:**
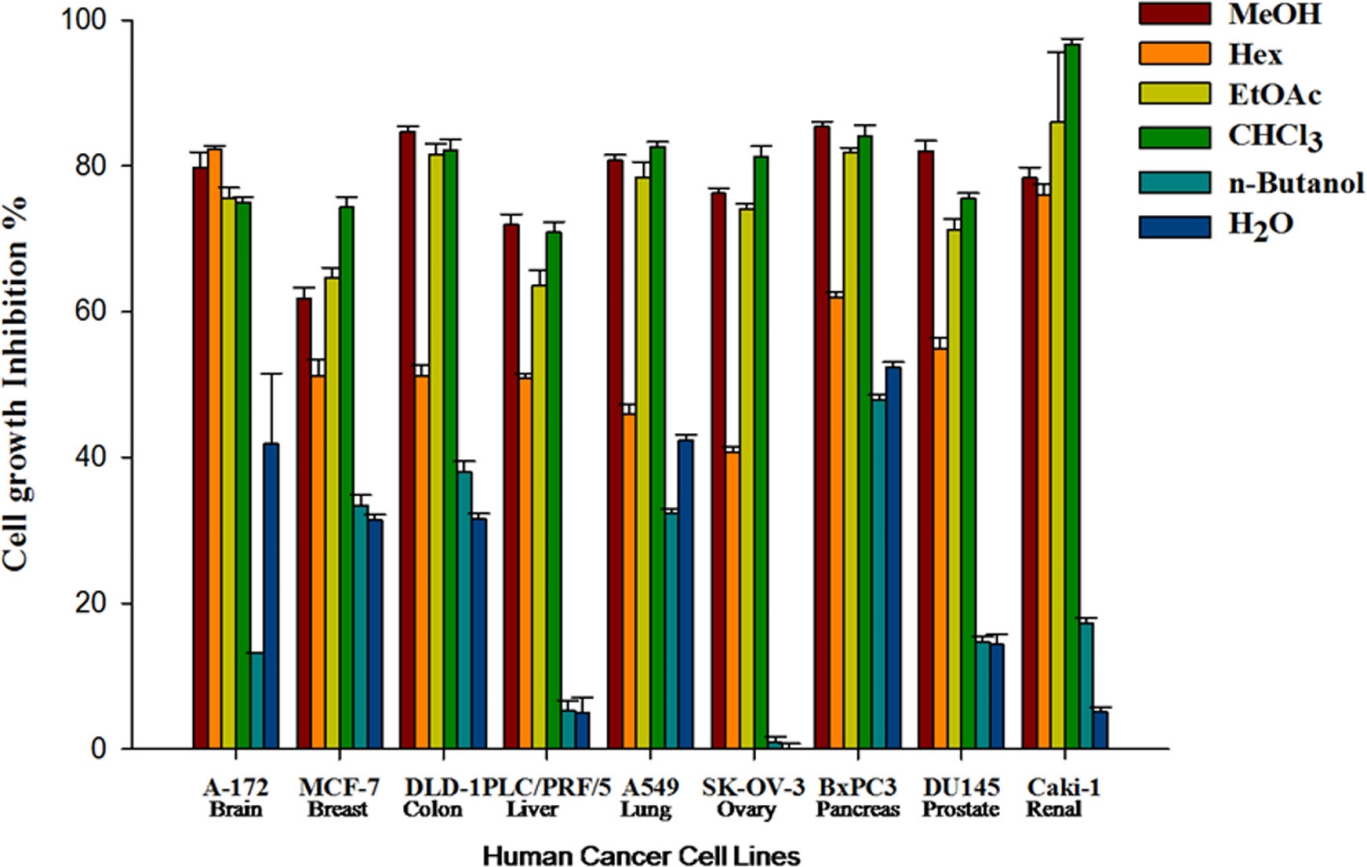
Percentage cell growth inhibition of solvent extracts of *Betula utilis* bark in nine different cancer cell lines.

*Betula utilis* bark was extracted in methanol and fractionated with n-hexane, chloroform, ethyl acetate and n-butanol and water. All the extracts were screened primarily for *in vitro* cytotoxic activity against nine different human cancer cell lines (A172- Glioblastoma, MCF-7 -Breast adenocarcinoma, DLD-1- Colorectal adenocarcinoma, PLC/PRF/5- Liver hepatoma, A549-Lung carcinoma, SK-OV-3- Ovarian carcinoma, BxPC-3- Pancreatic adenocarcinoma, DU145- Prostate carcinoma, and Caki-1- Renal carcinoma) by SRB assay[13, 14]. Methanolic, ethyl acetate and chloroform extracts were found to have significant activity in comparison to other extracts (Fig 1). As ethyl acetate extract found most suitable on the basis of cytotoxic activity as well as extractive yield, we have selected this fraction to isolate pure molecules.

### Isolation of triterpenes from ethyl acetate fraction of *Betula utilis* bark

**Fig 2:**
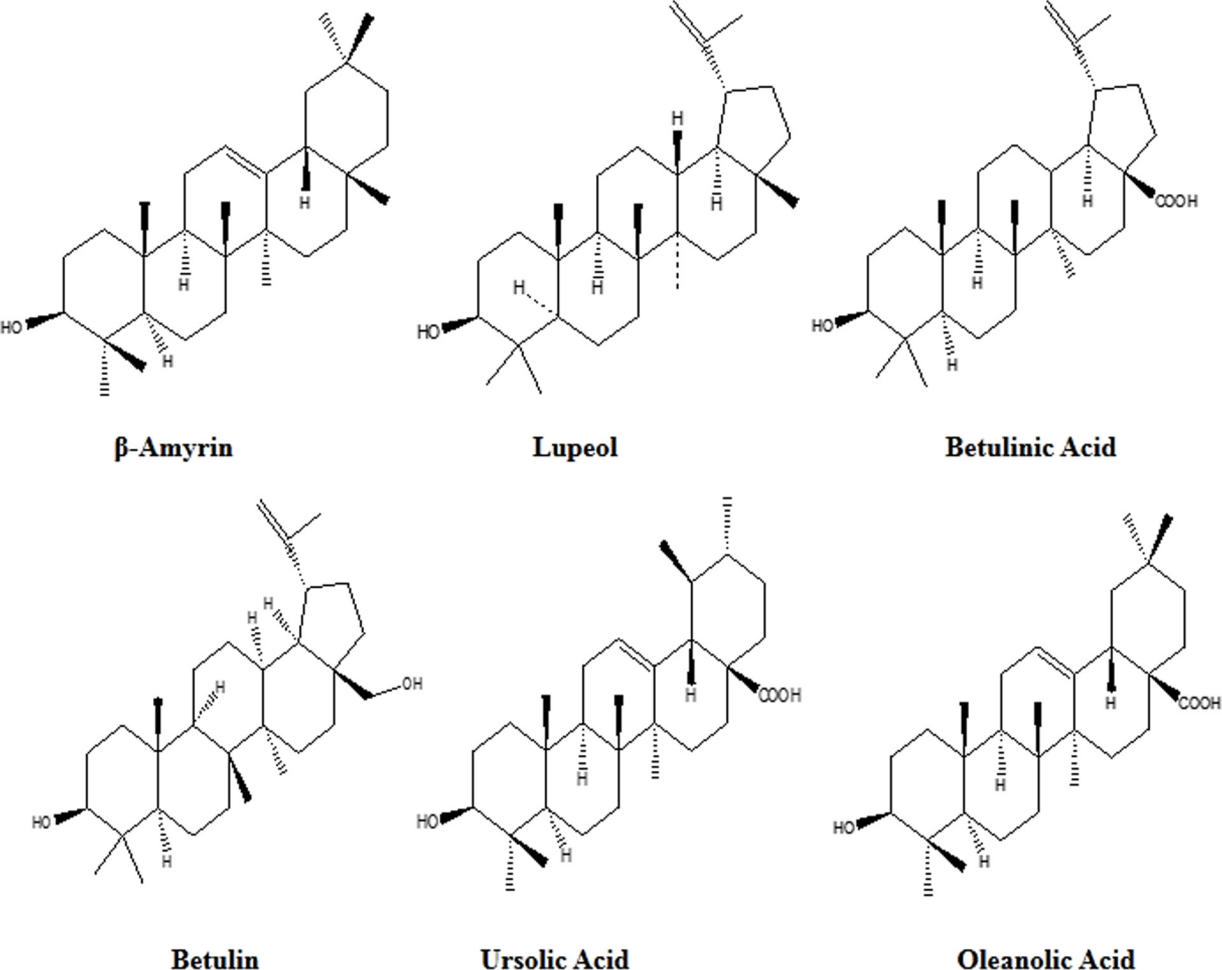
Chemical structures of isolated triterpenes from ethyl acetate fraction of *Betula utilis* bark.

The ethyl acetate fraction was column chromatographed over silica to obtain pure compounds (Fig 2). Isolated pure compounds were identified by means of spectroscopic analysis, and they were identified as β-Amyrin (1), Lupeol (2), betulinic acid (3), Betulin (4), Ursolic Acid (UA) (5), Oleanolic Acid (6). Out of these six compounds, β-Amyrin and UA were isolated for the first time from the bark of *Betula utilis*. β-Amyrin (1) resulted as a white amorphous solid and its mass spectrum exhibited molecular ion peak at m/z 426, with the molecular formula C_30_H_50_O. UV spectroscopy signifies absorption bands at 245 nm for conjugated double bond which is characteristic band, moreover IR spectra of compound 1 showed frequencies at 3290 cm^_1^ and 3036 cm^_1^ indicating the presence of hydroxyl group. MS spectra show m/z (relative intensity %) value at 218, 203, 207, and 189 which were attributed to pentacyclic triterpene amyrin. Proton NMR spectroscopy shows methyl singlet at 0.86, 0.89, 1.69 and olefinic proton resonating at d 5.46 (1H, t, J = 4 Hz, H-12). The ^13^C NMR spectra showed thirty carbons, two olefinic carbons at δ 121.11 (C-12) and 140.05 (C-13), along with Signal at δ72.91 showed presence of hydroxyl group which were the characteristic signals of the compound **(1)** [15]. All the spectroscopic details indicated that it is a pentacyclic triterpenes with molecular formula C_30_H_50_O. The chemical structure of identified compound **1** is given in Fig 2. UA **(5)** was obtained as white powder, its mass spectrum exhibited molecular ion peak at m/z 456. The molecular formula of UA (5) is C_30_H_48_O_3_. UV spectroscopy showed absorption band at 210 nm which is characteristic in nature. IR spectra of compound 5 shows presence of hydroxy group at 3434 cm^_1^ frequency and presence of carbonyl group at 1691 cm^_1^. Proton NMR shows methyl singlet 0.72, 0.76, 0.81, 0.90, 0.93, 0.94 and 1.05. The characteristic signal of ^13^C NMR spectra consisted at δ 181.1 because of carboxylic acid at C-28. There is two olefinic carbon, which gave signal at δ 126.02(C-12), 139.1(C-13) indicating urs-12-ene triterpenoid. MS: m/z (relative intensity %) 439, 248, 203, 189 and 119 [16]. The chemical structure of identified compound 5 is given in Fig 2. It is known that the β-amyrin and UA is widely distributed in the plant kingdom, however, to the best of our knowledge, the isolation and characterization of these triterpenes from *Betula utilis* has not been reported earlier. All the isolated triterpenes were identified on the basis of UV, IR and 1H NMR data and compared and validated with the existing literature.

### Cytotoxic activity of isolated triterpenes

**Fig 3:**
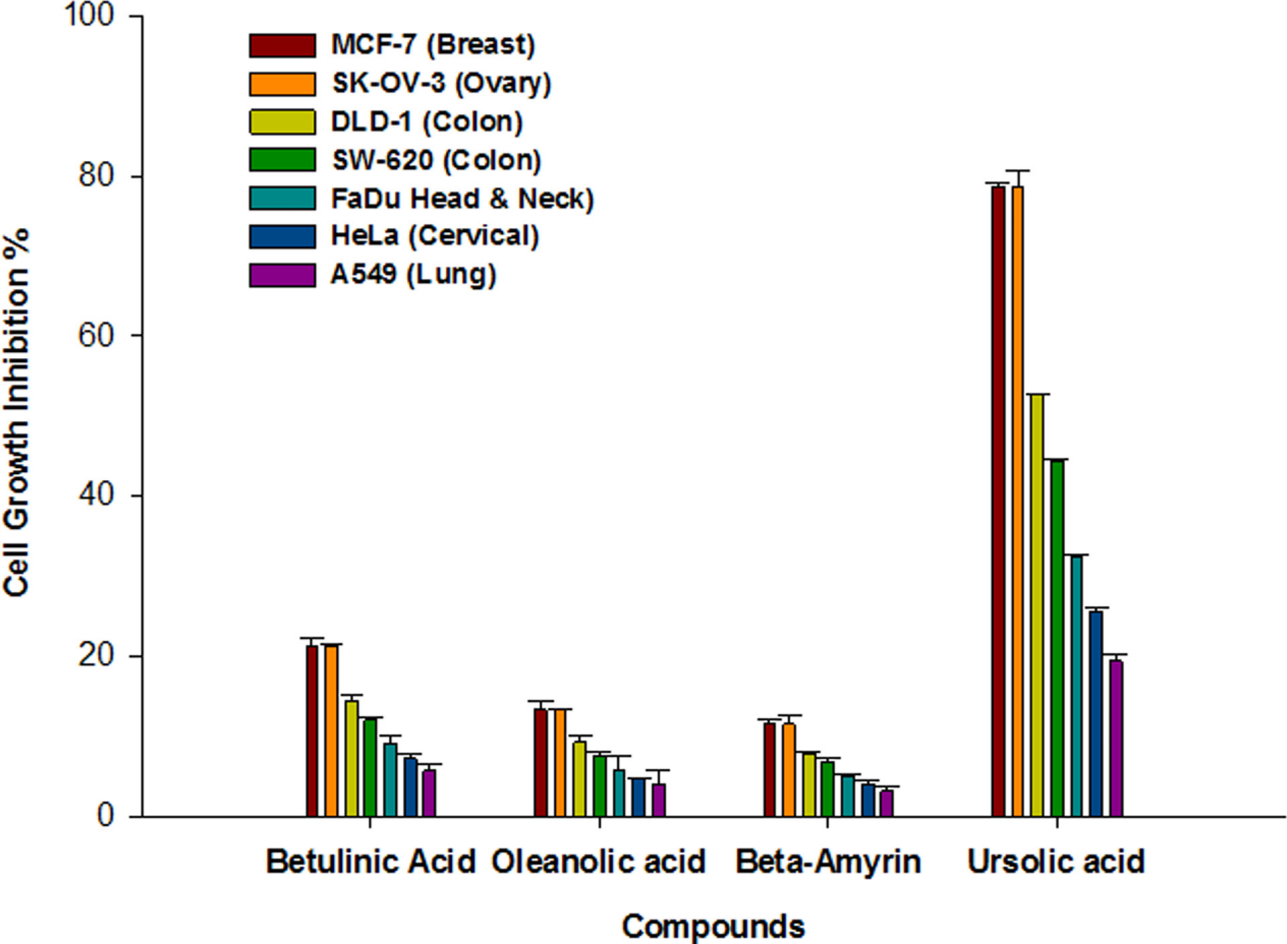
Cytotoxic activity of Isolated Triterpenes (10μM) against seven human cancer cell lines.

Out of six isolated triterpenes betulinic acid, oleanolic acid, beta amyrin and UA were found to be soluble, whereas betulin and lupeol were insoluble in DMSO. So we tested these four soluble triterpenes for *in vitro* cytotoxic activity against seven human cancer cell lines *viz*., breast (MCF-7), colon (DLD-1, SW620), lung (A549), ovary (SK-OV-3), Head and Neck (FaDu) and cervical (HeLa). The percentage inhibition has been shown in Fig 3. Out of four isolated triterpenes UA was found to be cytotoxic against different cancer cell lines but shows most significant cytotoxic activity in breast cancer.

### Tumor cell selective cytotoxicity of ursolic acid

**Fig 4:**
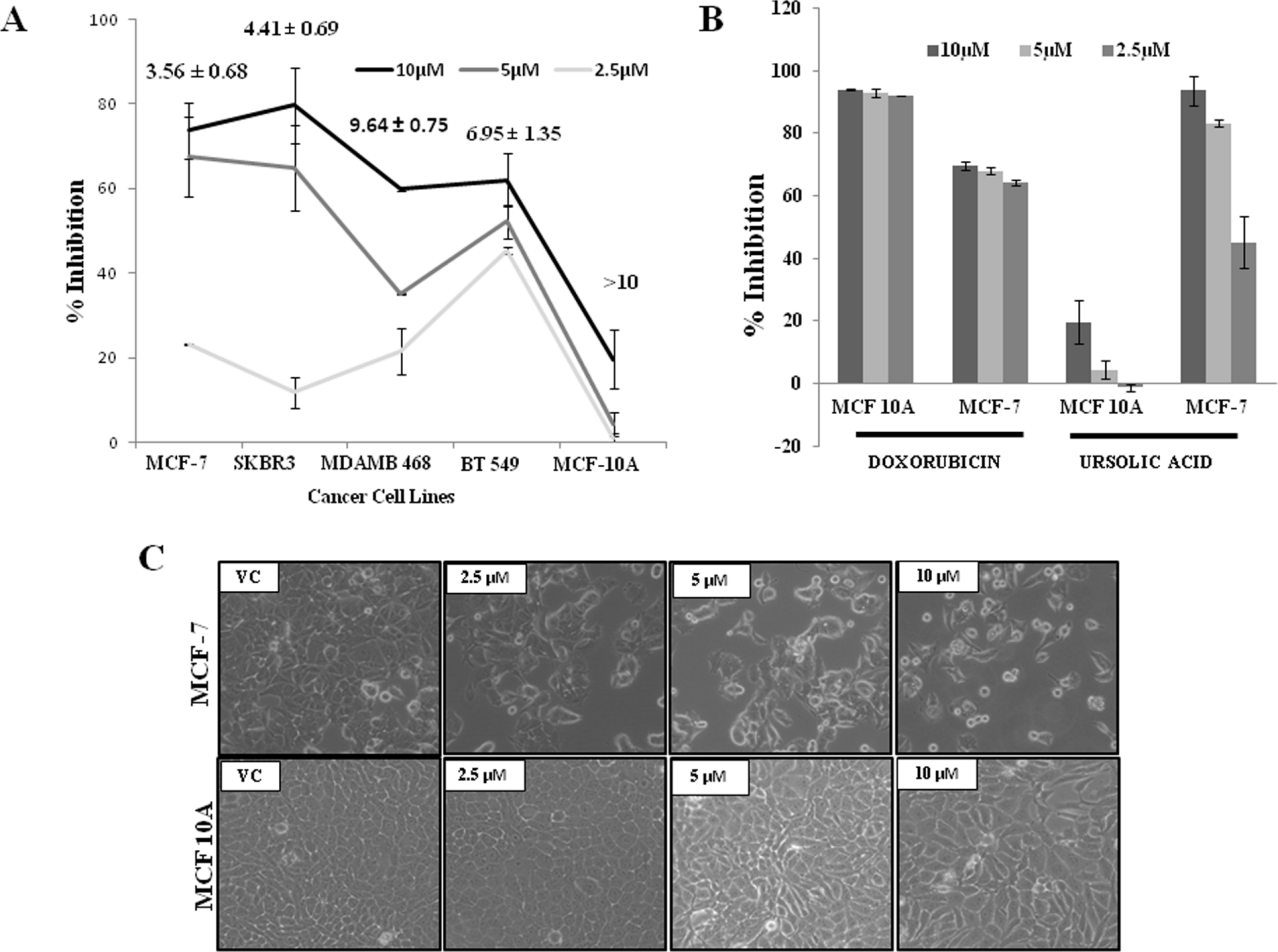
Selective Cytotoxicity of UA to tumorigenic MCF-7 over non tumorigenic MCF 10A. Percentage growth inhibition and IC_50_ of UA in different breast cancer cell lines. MCF-7 and MCF 10A cells were treated with either doses of 2.5, 5 and 10μM UA or Doxorubicin for 48 hours and cytotoxicity was assessed by SRB assay. Columns, average of triplicate readings of samples; error bars, ± S.D. **C.** Phase-contrast photomicrograph of MCF-7 and MCF 10A cells treated with either vehicle control or different doses of UA.

As UA is most active in breast cancer, we checked its cytotoxicity by standard SRB assay in four different breast cancer cell lines MCF-7, SK-BR-3, MDA-MB-468, BT-549 & one non tumorigenic breast epithelial cell line (MCF10 A). These cells were treated with increasing concentration of UA for 48 hours then percentage inhibition and Inhibitory concentration(IC)50 (Fig 4A) was calculated, here we found that UA is selective toxic to breast tumor cells with its maximum cytotoxicity to MCF-7 with relatively low cytotoxicity towards MCF-10A cells. To compare the efficacy of UA from *Betula utilis* bark with an existing drug, we treated MCF-7 and MCF 10A cells with UA and standard drug Doxorubicin and observed that Doxorubicin was found to be more cytotoxic in MCF 10A cells as compared to MCF-7 cells. In contrast, UA has shown minimal cytotoxicity against MCF 10A cells but it poses marked cytotoxic effects to MCF-7 cells (Fig 4B). In the presence of UA the non- tumorigenic cells grew normally, whereas the breast cancer cells underwent a change in morphology into spherical forms. Bright-field microscopy images of MCF-7 AND MCF10A cells show the morphological differences between vehicle and UA treated cells (Fig 4C). Columns, average of triplicate readings of samples; error bars, ± S.D.

### Apoptosis analysis

**Fig 5:**
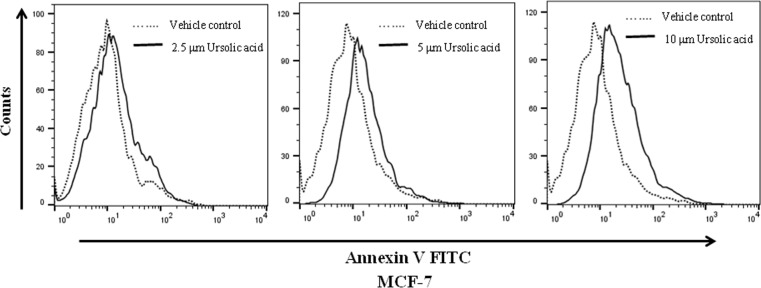
Apoptosis determined by Annexin V fluorescein isothiocyanate (AV-FITC) assay in MCF-7 cells after incubation with 2.5 μM, 5 μM and 10 μM of UA for 24 h.

To validate whether UA induces apoptosis in breast cancer cells, we performed Annexin-V staining to detect early apoptotic cells and analyzed by flow cytometer. In MCF-7 cells, UA treatment (24h) dose dependently increased early apoptotic cells compared to vehicle treated cells as indicated by the right shift of the histogram overlays (Fig 5). Results are representative of three independent experiments.

### Ursolic acid selectively promotes extrinsic apoptotic pathway in breast cancer

**Fig 6:**
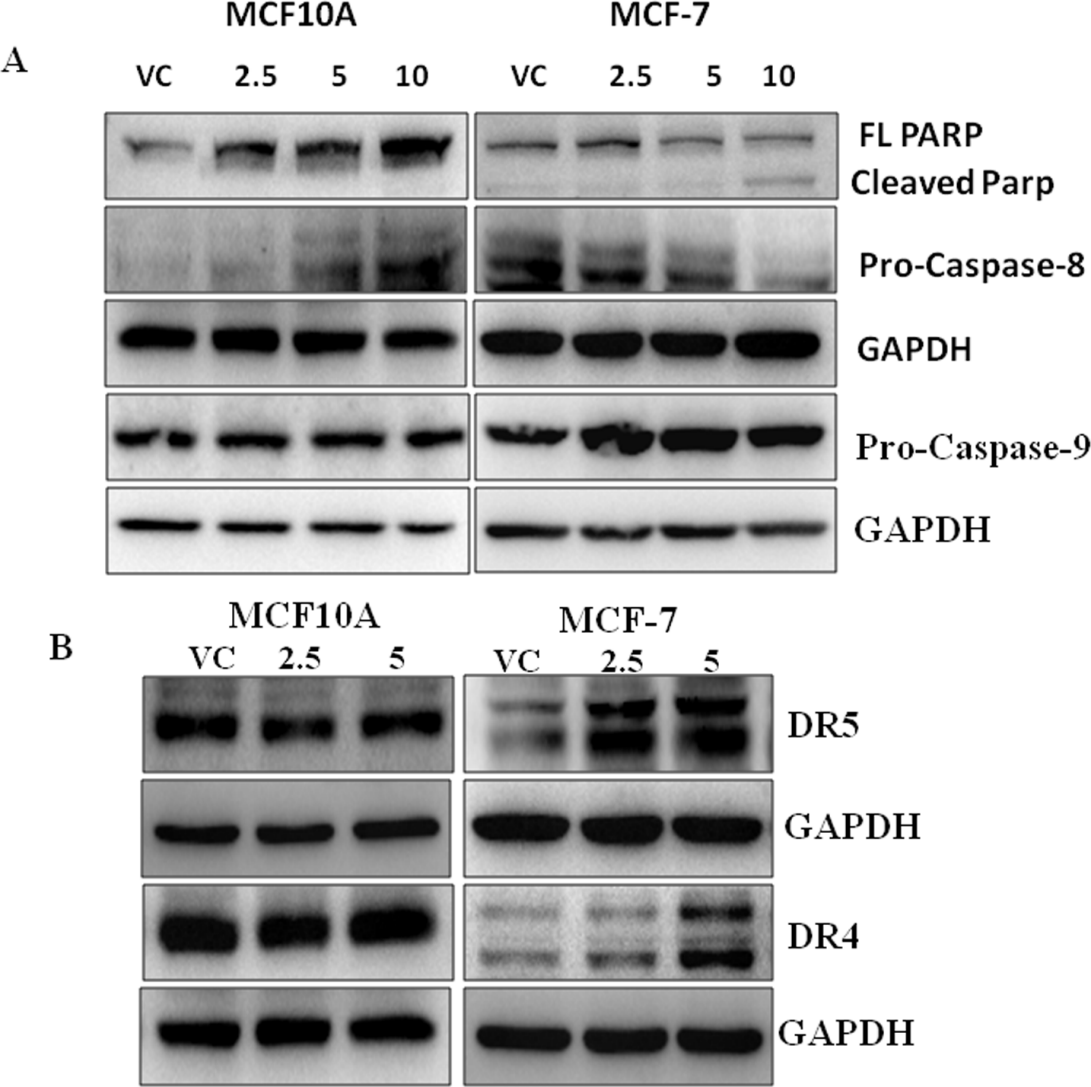
**A.** Immunoblot analysis for caspase-8, caspase-9 and PARP expression in MCF-7 and MCF10A cells (treated with the indicated concentrations (0, 2.5, 5 and 10μM of UA). GAPDH was used as loading control.**B.** Western Blot analysis for DR4 and DR5 expression in MCF-7 and MCF10A cells treated with the indicated concentrations (0, 2.5 and 5μM of UA). GAPDH was used as the loading control.

PARP cleavage is a biochemical hallmark of apoptosis. To test the cancer cell specific cytotoxicity of UA, we assessed the expression of PARP in tumorigenic transformed human breast adenocarcinoma cells MCF-7 and non-tumorigenic non-transformed breast epithelial cells MCF 10A by Western blot analysis. Here, interestingly we observed significant PARP cleavage after UA treatment compared to vehicle treatment in MCF-7. In contrast to MCF-7, we observed increasing full length PARP instead of cleaved PARP in MCF 10A, which indicated the selective cytotoxic effect of UA on tumorigenic MCF-7 over non-tumorigenic MCF 10A (Fig 6A) cells.

Caspases are the key downstream modulators of any apoptotic process and particularly, activation of caspase-8 and caspase-9 are classical discriminators of the extrinsic and intrinsic apoptosis pathways respectively. To check the involvement of either or both apoptotic pathway after UA treatment, we tested pro-caspase-8 and pro-caspase-9 expression in both MCF-7 and MCF 10A cells by western blot analysis. Here, we observed that UA treatment preferentially downregulated the expression of pro-caspase-8 in MCF-7 cells whereas, slightly increased in MCF10A cells compared to vehicle treatment, while pro-caspase-9 expression remained unaltered in both the cells (Fig 6A). Altogether, changes observed after UA treatment are tumor cell selective and indicative for the involvement of the extrinsic apoptotic pathway in breast cancer cells.

### Ursolic acid selectively induces the expression of death receptors DR4 and DR5 in MCF-7 cells but not in MCF 10A cells

The extrinsic pathway activates caspases via augmenting the expression of death receptors. To explore the underlying mechanism that may be responsible for the apoptotic effect of UA in breast cancer, we examined the expression of death receptors DR4 and DR5 in both MCF-7 and MCF 10A after UA treatment. Here, we observed that UA induced both DR4 and DR5 in a dose-dependent manner in MCF-7 but their expressions in MCF 10A remains unchanged (Fig 6B). Therefore, our results suggest that the selective upregulation of DR4 and DR5 in MCF-7 cells may be amenable for UA induced cytotoxicity/apoptotic effect in MCF-7 over MCF 10A cells.

### Ursolic acid generates Reactive oxygen Species (ROS) via H_2_O_2_ and disrupts the Mitochondrial Membrane Potential (MMP)

**Fig 7:**
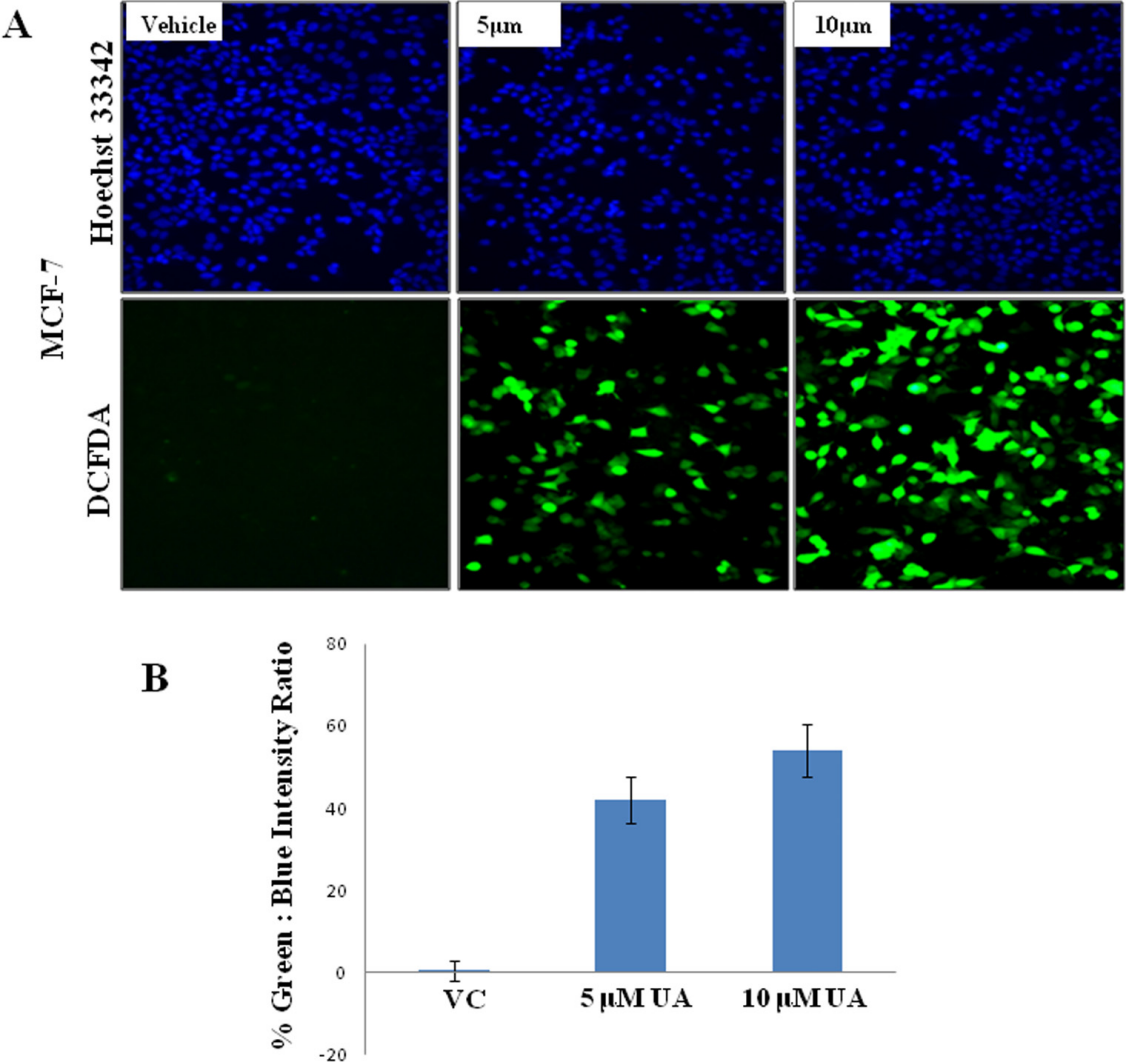
The effect of UA on ROS generation was determined using DCF-DA assay in Fluorescence Microscopy. **A.** Representative Fluorescence micrographs depicting ROS generation in MCF-7 cells after treatment with UA at 5μM and 10μM for 24 h. **B.** % of green DCFDA positive cells over total (DAPI stained) number of cells were calculated in 3 different fields of control and UA treated cells and represented in bar diagram; *bars*, +/− SD of control and treated groups.

**Fig 8:**
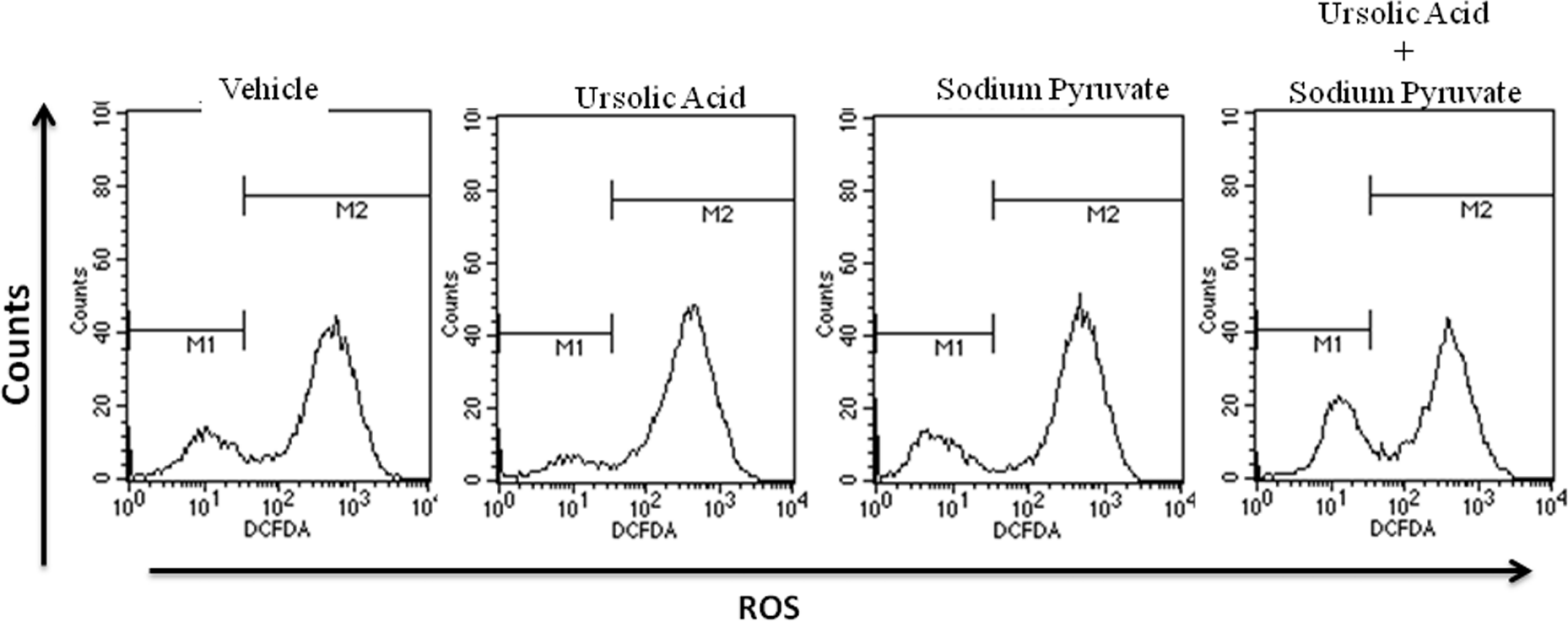
Representative flow cytometric plots showing the ROS generation in MCF-7 cells after the treatment of Vehicle, 5 μM UA, 1mM Sodium Pyruvate and 5 μM UA plus 1mM Sodium Pyruvate for 24 hours.

**Fig 9:**
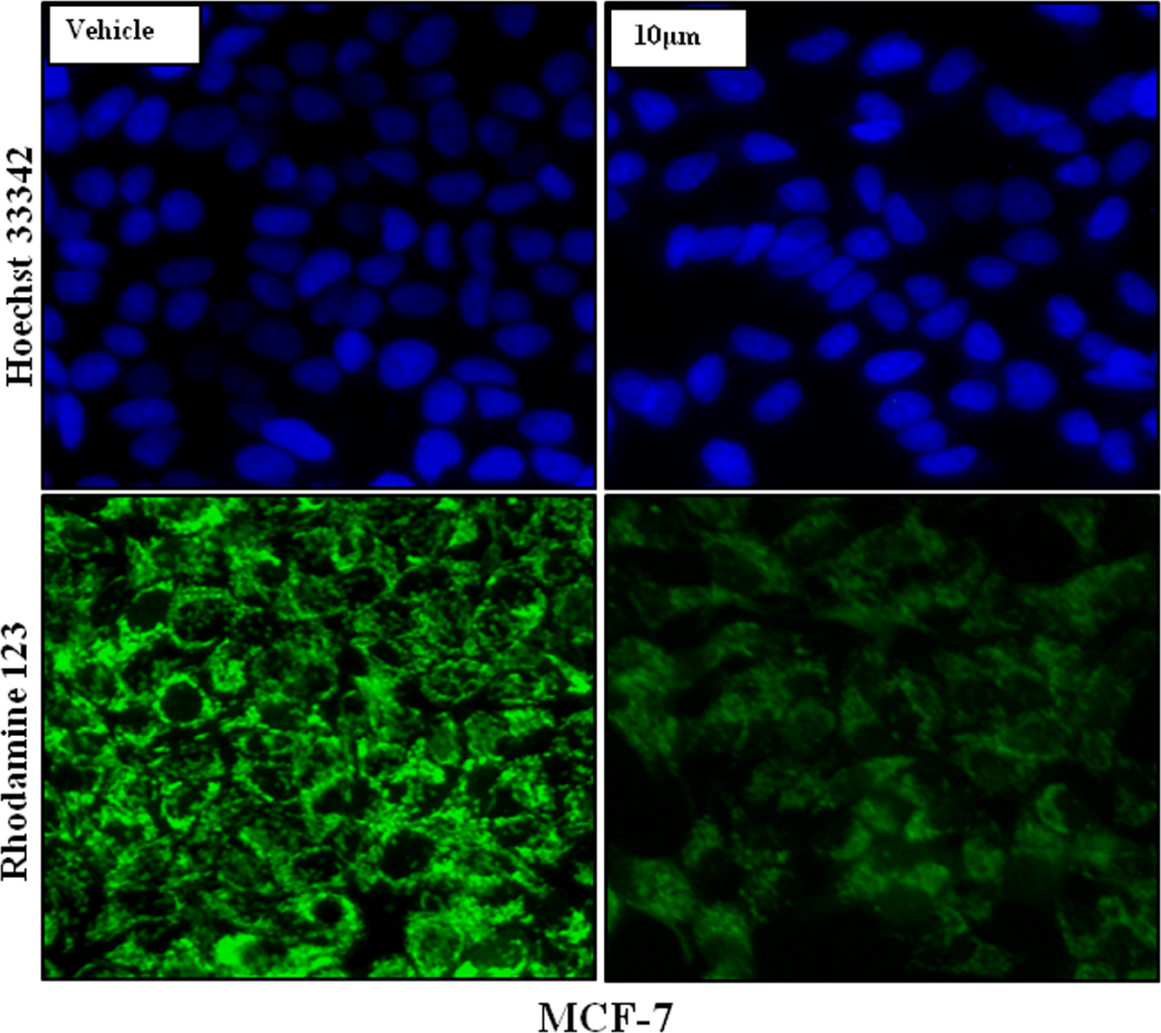
Mitochondrial Membrane Potential (MMP) disruption in MCF-7 cells treated by UA at 10 μM for 24 h.

Targeting the mitochondria to sensitize cancer cells to apoptosis is a major key for cancer cell death, to determine whether the anti-proliferative effect of UA was mediated by the generation of reactive oxygen species (ROS), the ROS level in MCF-7 cells was measured using DCFH-DA using fluorescent microscope (Fig 7A). Compared to untreated control cells, the ROS levels in MCF-7 cells were increased significantly after treatment with UA at various concentrations for 24 h. For quantitative analysis percentage of green: blue intensity ratio is calculated by Image J software (NIH, USA) and represented in bar diagram as shown in panel (Fig 7B). To dissect the mechanism via which UA is generating ROS, we treated MCF-7 cells with either UA or UA + sodium Pyruvate (Hydrogen peroxide inhibitor) or UA+ Diphenyleneiodonium (DPI) (NADPH Oxidase inhibitor) and found that the treatment of UA with sodium pyruvate was able to minimize the ROS generation in treated cells (Fig 8), while DPI was unable to quench the ROS generated by UA (data not shown). Decreased fluorescence of Rhodamine123 indicated a decrease in MMP and a loss of mitochondrial membrane integrity. Fluorescent analysis results from the treated/untreated MCF-7 cells were shown in Fig 9. Compared with the untreated group, levels of intracellular green fluorescence in UA incubated groups were gradually decreased after the treatment.

### Ursolic acid Inhibition of migration of human breast cancer cells *in vitro*

**Fig 10:**
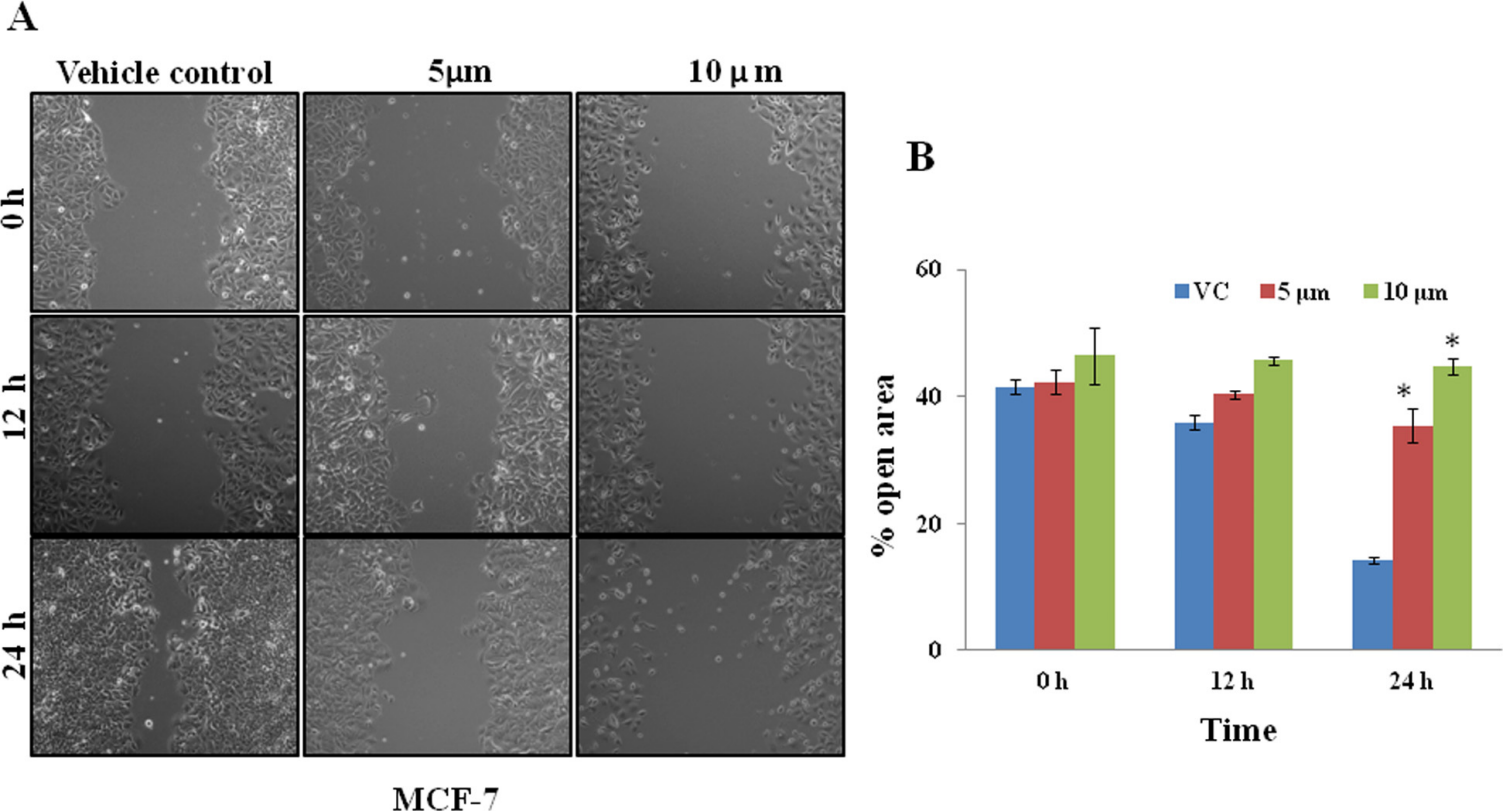
**A.** Inhibition of MCF-7 cells migration by UA (Pictorial representation)**. B.**Percentage of open area were shown in bar graph.

In order to investigate the effects of UA on the invasive potency of breast cancer cells, we carried out a wound-healing assay in MCF-7 human breast cancer cells. Monolayers of cells treated with UA at 5 and 10 μM concentrations shows inhibitory effect on MCF-7 cell migration at 12 and 24 h. (Fig 10A and 10B).

## Conclusion

The aim of the present study was to isolate bioactive compound from the bark of *Betula utilis*, which is responsible for anticancer activity. It is believed that cytotoxic property of *Betula utilis* bark was due to the presence of betulinic acid. Present study, however revealed that UA found more active than betulinic acid. Although UA have been evaluated for cancer prevention and treatment previously [17]. Besides significant *in-vitro* cytotoxic activity of UA for breast cancer, the selectivity of UA was tested against normal epithelial breast cancer cells MCF-10A cells. MCF-10A is a non-transformed and immortalized epithelial cell line originated from human fibrocystic mammary tissue. An ideal anticancer agent should be able to selectively target cancer cells but render no harm to normal cells. UA was relatively less cytotoxic to MCF-10A cells compared to MCF-7 cells at 48 hours as shown in Fig 4B. Hence, it indicates that the cytotoxic effect of UA was more selective towards breast cancer cells compared to the normal breast epithelial cells. Our data provide mechanistic evidence that UA treatment selectively activates extrinsic apoptosis pathway via upregulation of death receptors DR4 and DR5. Present study also demonstrated that MCF-7 cells undergo apoptosis in response to treatment with UA, which occurs through a mitochondrial-mediated pathway that requires ROS generation upstream to disrupt the MMP. Moreover UA showed a significant anti-migration potential in MCF-7 cells. Thus it can be concluded that cytotoxic activity of *Betula utilis* bark might be due to presence of UA, along with betulinic acid and oleanolic acid, and, therefore the present endeavour is a maiden attempt to unravel the anticancer potential of UA isolated from bark of the *Betula utilis* bark.

## Materials and Methods

### Plant material

The stem bark of the Himalayan Silver Birch (*Betula utilis*) along with voucher specimen (259039) was collected during June 2012 from the Govind Wildlife Sanctuary, Uttarakhand India. The samples were authenticated by the plant taxonomists at CSIR-National Botanical Research Institute, Lucknow, India.

### Extraction

Dried bark of the plant (3.0 kg) were milled into powder and then extracted with methanol (15 L) in an extractor for 36 h. The extract was evaporated in a rotatory evaporator and dried by vacuum pump. The methanolic extract (315 g) was suspended on water and extracted successively with hexane, ethyl acetate, chloroform, and butanol to yield hexane (19.77 g), chloroform (11.04 g) ethyl acetate (32.29g), and Butanol soluble (56.14 g) fractions, respectively.

### Reagents and antibodies

H_2_-DCFDA, Rh123, Doxorubicin, Sodium Pyruvate were obtained from Sigma Aldrich. PARP, Caspase-8, Caspase-9, DR4 and DR5 antibodies were obtained from Cell Signaling Technology, Inc. HRP-conjugated secondary antibodies were purchased from Santa Cruz Biotechnology. All chemicals and antibodies were obtained from Sigma unless specified otherwise.

### Cytotoxic activity

All the six extracts of *Betula utilis* were tested for *in vitro* cytotoxic activity against nine cancer cell lines. Ethyl acetate and chloroform extract were found to have most significant activity. Ethyl acetate extract was column chromatographed over silica gel for isolation of compound responsible for cytotoxic activity of *Betula utilis* bark.

### Cell culture and sample preparation

The human cancer cell lines such as Lung (A549), Colon (DLD-1,), Breast (MCF-7, MDA-MB-468), Head and Neck (FaDu), Cervical (HeLa), Prostate (DU145), Ovary (SK-OV-3), Brain (A-172), Liver (PLC/PRF/5), Pancreas (MIAPaCa-2), Renal (786–0, Caki-1) were maintained in RPMI-1640 medium, whereas other cell lines like Breast (MDA-MB-231) and Colon (SW 620) in DMEM medium. The test samples/ molecules weighed in micro-centrifuge tubes and stock solutions of 100 mg/mL were prepared by dissolving the samples in DMSO. Stock solutions were stored at -20°C. A working solution of 200 μg/mL was prepared by diluting the stock solution in culture medium (RPMI-1640 with 5% FBS) prior to the assay.

### Cytotoxicity assay (SRB assay)

A standard colorimetric SRB (sulforhodamine B) assay was used for the measurement of cell viability. Briefly, 10,000–20,000 cells (depending on the doubling time of each cell type) were seeded to each well of 96-well plate in 5% serum containing growth medium and incubated overnight in CO_2_ incubator at 37°C. Adhered cells were then treated with vehicle or fractions at the required dose. After 48 hours of exposure, cells were fixed with ice-cold 50% TCA, stained with 0.4% (w/v) SRB in 1% acetic acid, washed and air dried. Bound dye was dissolved in 10mM Tris base and absorbance was measured at 510 nm on a plate reader (Epoch Microplate Reader, Biotek, USA). The cytotoxic effects of the fractions were calculated as % inhibition in cell growth as per the formula [100-(Absorbance of treated cells/ Absorbance of vehicle treated cells)] X 100.[18].

During initial screening, the samples showing equal to or more than 75% growth inhibition of cancer cells at 10μm concentration were considered as ‘Hits’ and further screened at 2-fold serial dilutions against cancer cell lines to calculated their half maximal inhibitory concentration (IC_50_) value, and IC_50_ values were derived using Graph Prism software.

### Western blot analysis

Protein samples were run on 4–15% gradient SDS-polyacrylamide gel (BioRad) and transferred to a PVDF membrane (Millipore, USA). The membranes were incubated with different primary antibodies and subsequently incubated with peroxidase-linked appropriate secondary antibody. The protein expression was visualized by an enhanced chemiluminescence solution (ImmobilonTM western, Millipore, USA) and scanned by gel documentation system (Bio-Rad chemidoc XRS plus).

### Reactive Oxygen Species (ROS) generation and Mitochondrial Membrane Potential (MMP) disruption Study of ursolic acid in breast cancer

ROS generation and subsequent MMP disruptions are the keys for any apoptotic process. To test the role of UA in generating ROS and MMP disruption, MCF-7 cells were treated with the indicated concentrations of UA or sodium pyruvate for 24 h, then cells were treated with 25 μM dichloro-dihydro-fluorescein diacetate (DCFH-DA) for 30 min, and fluorescence was detected using a fluorescence microscope (Nikon-TI Microscope, Japan) or by utilizing flowcytometer. The experiments were repeated at least three times independently [19]. MMP was assessed using a fluorescent dye, Rh123, a cell-permeable cationic dye that preferentially enters the mitochondria based on the highly negative MMP. The depolarization of MMP results in the loss of Rh123 from the mitochondria and a decrease in intracellular green fluorescence. MCF-7 cells were cultured on 24 well plate and treated with 10 μm UA, the wells were washed 2 times with PBS then incubated with 1 mg/l Rh123 at 37°C for 30 min and washed briefly with PBS 2 times. Rh123 fluorescence was measured using a fluorescence microscope (Nikon-TI Microscope, Japan) [20]. This experiment was also carried out 3 times.

### *In-vitro* migration assay

Breast cancer cell migration was assessed by wound healing assay. Wound healing assay showing the inhibitory effect of 5 and 10 μM UA on MCF-7 migration for 12, and 24 h. Cells seeded into 12–well plates at 70–80% confluency. The wound was scratched with a 200ul pipette tip. Wound closure was monitored at the indicated time intervals and imaged with phase contrast microscopy on an inverted microscope (ziess Primo vert using a 10 x phase contrast objective). Mean ± SD, n = 3. (b). the % of open area (scratch) was quantified with TScratch software (ETH Zürich).

### Isolation of triterpenes

Ethyl acetate soluble fraction (30 g) was subjected to chromatography on silica gel (60–120 mesh, Qualigen) eluted with a step wise gradient of Hexane-EtOAc (9.5:0.5, 9:1, 8.5:1.5, 8:2, 7.5:2.5, 7:3) by volume to afford a total of 600 fractions of 100 ml each. Column fractions were analyzed by TLC, and fractions with similar TLC patterns were combined to give five major column fractions (Fraction-1, Fraction-2, Fraction-3, and Fraction-4). Column fraction-1 was washed with acetone and further purified by sephadex column chromatography to give two compounds as white amorphous solid (23.5mg) β-Amyrin (**1**) [21] and 18mg Lupeol **(2)** [22]. Column fraction 2 was purified by solvent washing to give white solid crystals (196 mg) Betulinic Acid (**3**) [23]. Column fraction 3 was further purified by sephadex column chromatography. Solvent system of sephadex column was (methanol-chloroform, 50:50) to give Betulin (**4)** (3.25gm) [22] and UA (**5)** as white powder (44 mg) [16]. Betulin was crystallized from ethanol as white needles. Column fraction 4 was further purified by preparative TLC over silica gel GF_254_ using methanol-chloroform (0.15:9.85) as developing solvent to get Oleanolic Acid (**6**). It was crystallized from methanol as white powder (13 mg). Spectroscopic Data UV, IR given as S1 Table whereas Proton and carbon NMR related to all six isolated triterpenes are given as S1 Table.

## Supporting Information

S1 Table UV (nm), IR (KBr) cm^-1^, Mass (M+) and M.P. (^0^C) data of isolated Triterpenes from *Betula utilis* bark. (DOCX)
