# Supplementary material for: Isolation, Characterization and Anticancer Potential of Cytotoxic Triterpenes from Betula utilis Bark
Source: PLoS One. 2016 Jul 25;11(7):e0159430. doi: 10.1371/journal.pone.0159430 (PMC4959718; doi:10.1371/journal.pone.0159430)
Supplement: S1 Table — (DOCX) [file pone.0159430.s001.docx]

**Supplementary Material to**

**Isolation, characterization and anticancer potential of cytotoxic Triterpenes from *Betula utilis* bark**

Tripti Mishra^# 1^, Rakesh Kumar Arya^#2^, Sanjeev Meena^#2^ Pushpa Joshi^3^, Mahesh Pal*^1^, Baleshwar Meena^4^, D. K. Upreti^4^ ,T. S. Rana^4^ and Dipak Datta*^2^. *^1^Phytochemistry Division, CSIR-National Botanical Research Institute, Lucknow 226 001, India^2^* *Biochemistry Division, CSIR-Central Drug Research Institute (CDRI), Lucknow-226031, India.*

^3^ *Department of chemistry, D.S.B. Campus Kumaun university, Nainital-*263002, India.

*^4^Plant Diversity, Systematics and Herbarium Division, CSIR-National Botanical Research Institute, Lucknow 226001, India*

# contributed equally to this work

*To whom correspondence should be addressed: Dr. Mahesh Pal, Phytochemistry Division, CSIR-National Botanical Research Institute, Lucknow, 226001, India, Email: [drmpal.nbri@rediffmail.com](mailto:drmpal.nbri@rediffmail.com) or Dr. Dipak Datta, Biochemistry Division, CSIR-CDRI, B.S. 10/1, Sector 10, Jankipuram Extension, Sitapur Road, Lucknow- 226031, India, Tel: 91-522-2772450 (Extn-4347/48), Fax: 91-522-2771941, E-mail: [dipak.datta@cdri.res.in](mailto:dipak.datta@cdri.res.in)

**S1 Table .** UV (nm), IR (KBr) cm^-1^, Mass (M+) and M.P. (^0^C) data of isolated Triterpenes from *Betula utilis* bark

| **S.N.** | **Compounds** | **M.P.** | **UV(nm)** | **IR (KBr)** **cm^-1^** |
| --- | --- | --- | --- | --- |
| **1.** | β-Amyrin (M^+^=426) | 187^0^C | 245 | 3292, 3035, 2964, 2945, 2850, 2717, 2669, 2032, 1753, 1660, 1460, 1385, 1309, 1262, 1128, 1030, 976, 882 |
| **2.** | Lupeol (M^+^=426) | 215 ^0^C | 350 | 3311, 2946, 2870, 1638, 1464, 1189, 1035, 998 |
| **3.** | Betulinic acid (M^+^=458) | 209 ^0^C | 206 | 3480, 3063, 2946, 2883, 2725, 1678, 1650, 1454, 1371, 1229, 1188, 1112, 1035, 978, 876, 871 |
| **4.** | Betulin (M^+^=442) | 255^0^C | 318 | 3386, 2953, 2865, 1649, 1552, 1384, 1032 |
| **5.** | Ursolic acid (M^+^=456) | 199 ^0^C | 210 | 3434, 3249, 3017, 2831, 2672, 2548, 1691, 1627, 1600, 1524, 1378, 1349, 1301, 1297, 1211, 963, 899, 845 |
| **6.** | Oleanolic Acid (M^+^=360) | 176^0^C | 209 | 3334, 3163, 3085, 2868, 2784, 2721, 2651, 1911, 1836, 1661, 1615, 1567, 1511, 1438, 1319, 1259, 1227, 1101, 1020, 961, 848, 810 |

NMR data of the all six triterpenes are given as follows:

**Beta Amyrin (1)**

^1^H NMR (δ values, CDCl_3_): 0.86 (s, 3H,-CH_3_), 0.89 (d, 3H, J=7Hz, H-30), 1.09 (d, 3H, J=7.0 Hz, H-27), 1.29 (d, 6H,J=11.5 Hz, H-18), 1.69 (s, 20H, -CH_3_×5, H-2, H-7 and H-5), 1.76 (m, 2H, H-19), 1.86 (m, 2H, H-22), 1.90 (m, 2H, H-15), 2.05 (d, 2H, J=11.5 Hz, H-1), 2.19 (s, 1H, H-9), 2.25 (d, 2H, J=11.5 Hz, H-1, J=11.5 Hz, H-1 and H-16), 5.21 (s, 2H, H-11), 5.46 (s, 1H, H-12). ^13^C-NMR (CD_3_OD 125MH_z_) : δ C 37.71 (C-1), 28.32 (C-2), 72.91 (C-3), 37.82 (C-4), 36.24 (C-5), 21.28 (C-6), 42.11 (C-7), 45.06 (C-8), 50.27 (C-9), 37.22 (C-10) 23.47 (C-11), 121.11 (C-12), 140.05 (C-13), 43.80(C-14), 26.47(C-15), 23.42 (C-16), 47.47 (C-17), 45.29 (C-18), 39.75 (C-19), 36.81 (C-20), 33.84 (C-21), 31.97 (C-22), 29.3 (C-23), 19.74 (C-24),19.16 (C-25), 18.96 (C-26), 18.35 (C-27), 19.04 (C-28), 36.24 (C-29), 19.19(C-30).

**Lupeol (2)**

^1^H NMR (δ values, CDCl_3_): 0.69 (d, 1H, J=9.1 Hz, H-5), 0.98 (s, 3H, H-23),0.91 (t, 1H,H-18) 0.84 (s,3H, H-25), 0.97 (s, 3H, H-27), 1.01 (d, 1H,H-15) 1.39 (q, 1H,H-6), 1.29 (q1H, H-12), 1.28 (s 1H H-9), 1.69 (s, 3H, H-30), 1.91 (m, 1H H-21), 2.37 (m 1H, H-19), 3.16 (dd, 1H, J=10.8, 5.1 Hz, H-3), 4.55 (br s, 1H, H-29). ^13^C-NMR (CD_3_OD 125MH_z_) : δC 38.71 (C-1), 27.32 (C-2), 78.90 (C-3), 38.86 (C-4), 55.24 (C-5), 18.28 (C-6), 34.11 (C-7), 41.06 (C-8), 50.28 (C-9), 37.22 (C-10) 21.47 (C-11), 25.12 (C-12), 38.05 (C-13), 42.80(C-14), 27.47(C-15), 35.52 (C-16), 42.97 (C-17), 48.21 (C-18), 47.95 (C-19), 150.83 (C-20), 29.84 (C-21), 39.93 (C-22), 27.93 (C-23), 15.34 (C-24),16.16 (C-25), 15.96 (C-26), 15.55 (C-27), 18.00 (C-28), 109.21 (C-29),19.17(C-30).

**Betulinic Acid (3)**

^1^H NMR (CDCl3): 0.67, 0.76, 0.99, 1.10 and 1.33 (5s, 15H, all tertiary –CH3), 1.39 (m, 2H, H-21), 1.40 (m, 2H, H-16), 1.47 (m, 2H, H-20), 1.53 (m, 4H, H-18, H-19 and H-15), 2.02 (m, 3H, H-1 and H-9), 2.17 (m, 2H, H-14), 3.25 (t, 2H, J =7 Hz, H-2), 3.36 (s, 2H, H-7), 4.59 (s, 2H, H-12), 4.55 (s, 2H, H-11). ^13^C-NMR (CDCl_3_, 125 MH_Z_), δ 39.7(C-1), 32.9(C-2), 76.8(C-3), 42.3(C-4), 60.1(C-5) 20.9(C-6), 45.0(C-7), 40.8 (C-8), 49.0(C -9), 32.4(C-10), 22.4(C-11), 26.02(C-12), 39.1(C -13)41.7(C-14), 31(C-15), 32.5(C-16), 57.6(C-17), 50.0(C-18), 48.4(C-19), 150(C-20),34.8(C-21), 33.5(C-22), 24.4(C-23), 20.1(C-24),16.6(C-25), 17.2(C-26), 15.5(C-27), 184.1(C-28),111(C-29), 20(C-30).

**Betulin (4)**

^1^H NMR (δ values, CDCl_3_): 0.68 (d, 1H,J=9.4 Hz, H-5), 0.75 (s, 3H, H-24) 0.80 (s, 3H, H-25), 0.99 (s, 3H, H-27), 0.96 (s, 3H, H-23), 0.97 (s, 3H, H-26), 1.66 (s, 3H, H-30), 2.31 (dt, 1H, J=10.5, 6.2 Hz, H-19), 3.16 (dd, 1H, J=10.8, 4.9 Hz, H-3), 3.33 (d, 1H, J=10.8, H-28), 3.79 (d, 1H, J=10.8 Hz, H-28), 4.58 (br s, 1H, H-29), 4.69(br s, 1H, H-29). ^13^C-NMR (CDCl_3_, 125 MH_Z_) , δ 38.7(C-1), 28.5(C-2), 78.8(C-3), 39.3(C-4), 56.3(C-5) 19.4(C-6), 35.4(C-7), 40.8 (C-8), 49.9(C-9), 36.9(C-10), 21.2(C-11), 26.02(C-12), 38.2(C-13), 41.9(C-14), 28(C-15), 28.9(C-16), 47.6(C-17), 50.0(C-18), 48.4(C-19), 150.5(C-20), 30.7(C-21), 33.8(C-22), 27.9(C-23), 16.0.1(C-24),16.6(C-25), 16.0(C-26), 15.3(C-27), 61.1(C-28),110(C-29), 20(C-30).

**Ursolic acid (5)**

^1^H NMR (CDCl3): 0.72, 0.76, 0.81, 0.90, 0.93, 0.94 and 1.05 (7s, 21H, all –CH3), 1.38 (m, 2H, H-21), 1.40 (m, 2H, H-16), 1.42 (m, 2H, H-20), 1.50 (m, 4H, H-18, H-19 and H-15), 2.07 (m, 3H, H-1 and H-9), 2.16 (m, 2H, H-14), 3.13 (t, 2H, J= 7 Hz, H-2), 3.34 (s, 2H, H-7), 4.53 (s, 2H, H-11), 4.58 (s, 1H, H-12). ^13^C-NMR (CDCl_3_, 125 MH_Z_), δ 38.7(C-1), 28.4(C-2), 77.8(C-3), 38.3(C-4), 56.12(C-5) 19.1(C-6), 33.05(C-7), 40.8 (C-8), 49.1(C-9), 36.4(C-10), 23.4(C-11), 126.02(C-12), 139.1(C-13), 41.9(C-14), 39(C-15), 23.5(C-16), 48.6(C-17), 52.8(C-18), 38.9(C-19), 39.4(C-20), 31.8(C-21), 36.9(C-22), 28.4(C-23), 16.1(C-24),16.6(C-25), 17.2(C-26), 24.0(C-27), 181.1(C-28),17.8(C-29), 21.0(C-30).

**Oleanolic Acid (6)**

^1^H NMR (CDCl3): 1H NMR (CDCl3): 0.75, 0.85, 0.91, 0.93, 0.97, 1.04 and 1.13 (7s, 21H, all –CH3), 1.35 (m, 2H, H-21), 1.39 (m, 2H, H-16), 1.51 (m, 5H, H-18, H-19 and H-15), 2.07 (m, 3H, H-1 and H-9), 3.12 (t, 1H, J = 7 Hz, H-2), 3.31 (s, 2H, H-7), 4.52 (s, 2H, H-11), 4.49 (s, 1H, H-12). ^13^C-NMR (CD_3_OD 125 MH_z_) : δ C 38.6(C-1), 27.0 (C-2), 78.4(C-3), 45.9(C-4), 55.1(C-5), 19.7(C-6), 33.5(C-7), 40(C-8), 46.5(C-9), 37.6(C-10) 23.9(C-11), 122.3(C-12), 143.68(C-13), 42.6(C-14), 28.5(C-15), 23.8(C-16), 47.3(C-17), 45.6(C-18), 46.8(C-19), 30.4(C-20), 34.8(C-21), 33.0(C-22), 25(C-23), 19.4(C-24), 16.2(C-25), 17.5(C-26), 26.5(C-27), 182.5 (C-28), 33.6(C-29), 23.8(C-30).
